# Pharmacotherapeutic Interventions for Sensorineural Hearing Loss: A Scoping Review

**DOI:** 10.3390/audiolres16030091

**Published:** 2026-06-14

**Authors:** Matthew Mavandi, Jack Hyler, Eric Lee, Ramanjot Singh, De Wet Swanepoel, Ashley M. Nassiri, Vinaya Manchaiah

**Affiliations:** 1 College of Osteopathic Medicine, Kansas City University, Kansas City, MO 64106, USA; matthew.mavandi@kansascity.edu (M.M.); eric.lee@kansascity.edu (E.L.); ramanjot.singh@kansascity.edu (R.S.); 2 Helzberg School of Management, Rockhurst University, Kansas City, MO 64110, USA; 3 Department of Speech-Language Pathology and Audiology, University of Pretoria, Pretoria 0001, South Africa; dewet.swanepoel@up.ac.za (D.W.S.); vinaya.manchaiah@cuanschutz.edu (V.M.); 4 Department of Otolaryngology-Head and Neck Surgery, University of Colorado Anschutz School of Medicine, Aurora, CO 80045, USA; 5 Virtual Hearing Lab, Collaborative Initiative University of Colorado School of Medicine, Aurora, CO 80045, USA; 6 Virtual Hearing Lab, Collaborative Initiative, University of Pretoria, Pretoria 00001, South Africa; 7 UCHealth Hearing and Balance, University of Colorado Hospital, Aurora, CO 80045, USA; 8 Department of Speech and Hearing, School of Allied Health Sciences, Manipal Academy of Higher Education, Manipal 576104, India

**Keywords:** hearing loss, sensorineural hearing loss, pharmacotherapy, antioxidants, gene therapy, ototoxicity, clinical trials

## Abstract

Background/Objectives: Sensorineural hearing loss (SNHL) is a chronic condition with no established pharmacological treatment. Recent advances in drug-based therapies offer promising opportunities to prevent or treat SNHL. This scoping review summarizes the current landscape of pharmacotherapeutics for SNHL. Methods: This scoping review was conducted in accordance with the Preferred Reporting Items for Systematic Reviews and Meta-Analyses extension for Scoping Reviews (PRISMA-ScR). A literature search of PubMed, Google Scholar, Embase, Scopus, and Web of Science was conducted in 2024 using keywords related to SNHL and pharmacotherapeutics. A review protocol was preregistered on the Open Science Framework. A systematic search of five electronic databases identified published studies from 2004 to 2024 on pharmacological treatments for SNHL in human participants, as well as ongoing clinical trials. Interventions were categorized by mechanism of action: antioxidant therapy, steroid-based combination therapy, hematologic-based therapy, pathway modulator therapy, regenerative therapy, and gene therapy. A narrative synthesis approach was used to map key trends across treatment types, study designs, and outcomes. Results: Sixty-six records met the inclusion criteria, including 48 published studies and 18 ongoing or recently completed clinical trial records. Antioxidants, corticosteroids, hematologic agents, and pathway modulators have demonstrated potential in preventing or treating SNHL caused by cisplatin, aminoglycosides, noise-induced ototoxicity, and intraoperative cochlear implantation trauma. Emerging regenerative and gene therapies show promise as future pharmacologic treatment options. Conclusions: Pharmacologic therapies for SNHL are promising but remain constrained by small sample sizes, heterogeneous study designs, and drug delivery challenges across the blood–labyrinth barrier. Future research should prioritize multicenter randomized trials, optimized delivery strategies, and integration of precision medicine approaches.

## 1. Introduction

Sensorineural hearing loss (SNHL) is the most common type of permanent hearing loss, caused by damage to the inner ear or auditory pathways [1]. The World Health Organization estimates that by 2050, nearly 2.5 billion people will have some degree of hearing loss, and 700 million will require hearing rehabilitation services worldwide [2]. Unlike visible disabilities, hearing loss may be insidious and, if left untreated, may lead to social isolation, loneliness, frustration, and dementia [3]. SNHL may arise from a variety of causes, including genetic mutations, exposure to ototoxic substances, noise exposure, infectious and immune processes, aging, or idiopathic sudden hearing loss [4,5,6,7,8,9].

While most people with SNHL may benefit from hearing amplification, only one in four or five people with hearing loss use amplification devices [10]. In recent years, progress has been made in the field of pharmacotherapeutics for SNHL. Several pharmacologic approaches have been investigated, including antioxidants, corticosteroids, hematologic-based therapies, and gene therapies [11,12,13]. Despite these efforts, pharmacologic treatment for SNHL remains fragmented, with no universally accepted therapeutic approach. The variability in mechanisms of action, clinical trial designs, and patient populations underscores the need for a comprehensive review of ongoing pharmacotherapeutic research.

A recent systematic review provided an overview of pharmacologic interventions for SNHL, summarizing key investigational treatments and their mechanisms [14]. However, the review did not comprehensively categorize therapies based on their clinical applications—protection, prevention, treatment, and regeneration—nor did it assess the breadth of clinical trials currently underway. Furthermore, gaps in existing research, such as the lack of standardized outcome measures and limited long-term follow-up data, highlight the need for a scoping review to synthesize the available literature and identify promising areas for future investigation [15,16].

This scoping review maps the current landscape of pharmacological treatments for SNHL, either by protection, prevention, treatment, or regeneration. By categorizing interventions based on their mechanisms of action and clinical applications, this review aims to provide clinicians and researchers with a practical overview of existing and emerging therapeutic options for SNHL. Additionally, areas for further research and improvements in screening methodologies are discussed, with the goal of advancing knowledge for clinicians about current pharmacologic options for SNHL prevention and treatment.

## 2. Materials and Methods

### 2.1. Study Design

This scoping review was conducted and reported in accordance with the Preferred Reporting Items for Systematic Reviews and Meta-Analyses extension for Scoping Reviews (PRISMA-ScR) [17]. This framework was utilized to ensure a structured, comprehensive, and standardized approach to the methodology and reporting of findings. A review protocol was preregistered on the Open Science Framework (23 October 2024; embargoed registration) [18]. This study was exempt from Institutional Review Board review per the authors’ institutional policy on scoping reviews.

### 2.2. Eligibility Criteria

To be included in this review, articles needed to meet the following inclusion criteria:

▪ Population: Human participants of any age or sex, with congenital or acquired SNHL.
▪ Concept: Pharmacological treatments for SNHL, including approved drugs and investigational therapies (e.g., corticosteroids, gene therapies).
▪ Context: Studies conducted in any setting, published between 2004 and 2024.
▪ Study Types: Any study design including randomized controlled trials (RCTs), non-randomized controlled trials, pretest–posttest studies, interrupted time-series studies, prospective and retrospective cohort studies, case–control studies, analytical and descriptive cross-sectional studies, observational studies, and case series with more than five patients published in peer-reviewed journals. Ongoing clinical trials registered with ClinicalTrials.gov were included to characterize the active investigational pipeline; however, these trial records were not treated as equivalent to published efficacy evidence and were summarized separately.

### 2.3. Information Sources and Search Strategy

A systematic literature search was conducted across five electronic databases: PubMed, Scopus, Embase, Web of Science, and Google Scholar. Additional searches were conducted through manual screening of reference lists from all included studies to identify potentially eligible publications not captured through database searches. The search strategy was initially developed for PubMed and adapted for the other databases. The search strategy is provided in the Appendix A.

### 2.4. Screening Methods and Selection Process

Two authors conducted the electronic search and study selection (M.M., J.H.). Search results from all databases were exported to Rayyan^®^ review (Rayyan, Cambridge, MA 02142, USA) for deduplication and screening. Two independent reviewers (E.L., R.S.) conducted title and abstract screening using the inclusion and exclusion criteria. Discrepancies were resolved by discussion with other authors (D.W.S., A.M.N., or V.M.) to ensure quality control. Full texts of the included studies were subsequently reviewed for final eligibility and data extraction.

### 2.5. Data Extraction and Synthesis

Four authors (M.M., J.H., E.L., R.S.) utilized a standardized data extraction form to capture key details from included studies, such as: author and year; treatment type (e.g., corticosteroids, antioxidants, gene therapies); study design and population (e.g., number of patients, RCT); outcome measures (e.g., hearing improvement, adverse events). This data form was made using a structured form in Google Sheets^®^ (Google, Mountain View, CA, USA). Narrative synthesis was used to descriptively map trends in treatment modalities and outcomes of the included studies.

## 3. Results

### 3.1. Search Results

**Figure 1:**
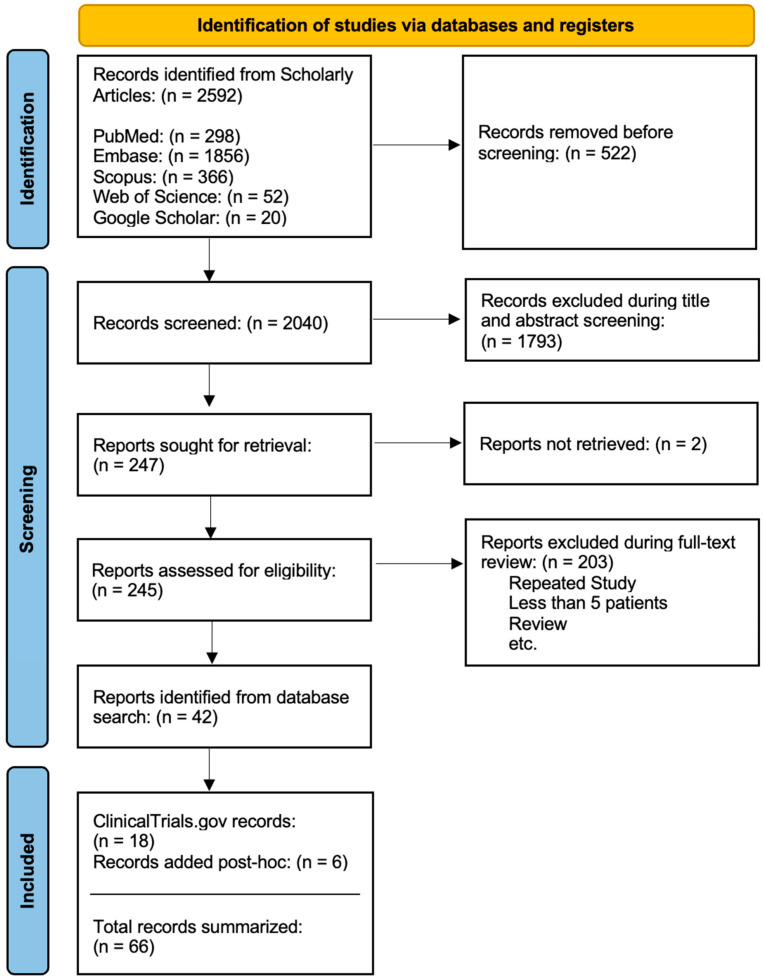
PRISMA-style flow diagram. This diagram depicts the methodology used to identify, screen, and include studies that were included in this review. No quantitative synthesis or meta-analysis was performed.

The initial search identified a total of 2592 articles: 298 from PubMed, 366 from Scopus, 1856 from Embase, 52 from Web of Science, and 20 from Google Scholar. After removing 552 duplicates, 2040 articles remained for title and abstract screening. Of these, 247 articles were selected for full-text review, and 42 met the inclusion criteria. In addition to database searching, manual searches were conducted to identify eligible studies not captured by the original search strategy. These included reference-list screening of included articles and targeted searches of ClinicalTrials.gov for ongoing pharmacotherapeutic trials relevant to SNHL. An additional 6 published studies and 18 ongoing clinical trials were identified through this process. To minimize selection bias, these records were screened using the same eligibility criteria as database-derived records, and reasons for inclusion were reviewed by the author team before incorporation into the final synthesis. Figure 1 provides further details about the search and screening process.

### 3.2. Summary of Included Studies

**Figure 2:**
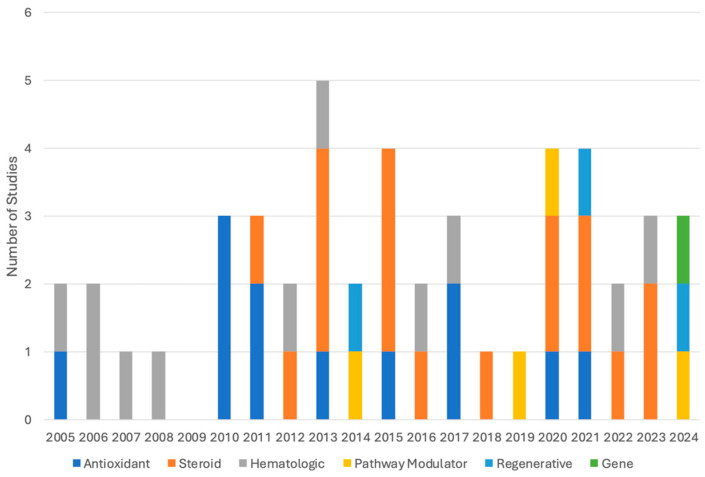
Number of studies per year graph. This stacked column graph depicts the number of studies per year by mechanism of action. Clinical trial records were excluded from this figure.

The review included 66 records: 48 published studies and 18 ongoing or recently completed clinical trial records. To organize the diverse pharmacological interventions identified for SNHL, a categorization framework was developed based on the primary therapeutic mechanism or target. The interventions were grouped by mechanism of action into the following categories: antioxidant therapy (*n* = 12), steroid-based combination therapy (*n* = 17), hematologic-based therapy (*n* = 11), pathway modulator therapy (*n* = 4), regenerative therapy (*n* = 3), and gene therapy (*n* = 1). An additional 18 ongoing or recently completed clinical trial records were included across all categories (*n* = 18). The section below provides further details on some of the key agents being studied. Table 1 summarizes all studied agents, which are organized by mechanism of action. Table 2 summarizes the 18 clinical trials sorted by mechanism of action. Figure 2 is a stacked column graph that illustrates the distribution of included published studies by year and mechanism of action over the 2004–2024 study period. Clinical trial records were excluded from this figure to avoid combining completed published evidence with ongoing or unpublished investigations. Overall, the trend demonstrates a gradual expansion of pharmacotherapeutic research for SNHL, with earlier studies primarily focused on steroid-based combination, antioxidant, and hematologic-based therapies, while more recent years show increasing attention toward pathway modulator, regenerative, and gene-based therapies.

### 3.3. Antioxidant Therapy

Table 3 summarizes twelve studies investigating antioxidant therapies for SNHL, encompassing six different agents. Antioxidants have been increasingly explored for their potential to mitigate SNHL by preventing oxidative stress-induced cellular damage from ototoxic insults such as chemotherapy, aminoglycosides, and noise exposure. One of the most notable advancements is the Food and Drug Administration (FDA) approval of PEDMARK as a preventative treatment for hearing loss. PEDMARK is specifically indicated for pediatric patients with localized, non-metastatic solid tumors undergoing platinum-based chemotherapy. PEDMARK is a highly purified, injectable formulation of sodium thiosulfate (STS) designed to reduce the ototoxic effects of cisplatin, a chemotherapeutic agent widely used in pediatric oncology [37]. When administered intravenously six hours after cisplatin infusion, STS selectively binds to and detoxifies reactive platinum species in non-cancerous tissues, particularly the cochlea. This delayed administration strategy reduces systemic and cochlear toxicity without compromising the anti-tumor efficacy of cisplatin [38]. The ACCL0431 study and the SIOPEL-6 trial both demonstrated that delayed infusion of STS significantly reduced the incidence and severity of cisplatin-induced hearing loss in pediatric patients [37,38,39].

Beyond STS, several other antioxidant agents are under active investigation. ORC-13661, a new chemical entity (NCE), is undergoing a phase 2 clinical trial as a potential otoprotective agent against aminoglycoside-induced ototoxicity [20]. SPI-1005, an oral formulation of the selenium-based compound ebselen, is also currently being evaluated in a phase II clinical trial for its ability to preserve residual hearing during cochlear implant surgery [19]. Ebselen mimics and enhances the activity of glutathione peroxidase (GPx), a critical antioxidant enzyme in the cochlea that detoxifies reactive oxygen species. Kil and colleagues have led multiple clinical trials investigating its potential to treat or prevent hearing loss in Meniere’s disease, cisplatin-induced ototoxicity, aminoglycoside-induced hearing loss, and noise-induced hearing damage [52,53,54].

Other widely studied compounds include N-acetylcysteine (NAC), Coenzyme Q10, and various vitamin-based regimens. Multiple clinical trials attempted to characterize the protective effects of NAC on either cisplatin-, aminoglycoside-, or noise-induced ototoxicity, but its efficacy has not met initial expectations [42,43,44,45]. Coenzyme Q10 treatment for presbycusis (age-related SNHL) resulted in modest but statistically significant improvements in audiometric thresholds at 1000, 2000, 4000, and 8000 Hz [46,47,48]. Vitamin-based therapies such as ACEMg and high-dose vitamin C combinations have demonstrated protective effects against oxidative stress in cochlear tissues, particularly in cochlear implant patients [49,50,51]. Taken together, these findings suggest that antioxidant therapy remains a promising but clinically heterogeneous approach, with the strongest current evidence supporting sodium thiosulfate for cisplatin-induced ototoxicity prevention.

### 3.4. Steroid-Based Combination Therapy

Corticosteroids remain the first-line therapy for sudden sensorineural hearing loss (SSNHL), largely due to their anti-inflammatory and immunosuppressive properties, which help limit cochlear damage. Table 4 summarizes seventeen studies that have investigated alternative delivery methods and complementary therapies to enhance steroid efficacy. For example, Dispenza et al. (2013) reported a mean 12.8 dB hearing improvement with intratympanic dexamethasone in patients unresponsive to systemic therapy [6]. CI522D is a newly developed cochlear implant designed to slowly release dexamethasone to reduce the trauma associated with cochlear implantation [21]. In comparing prednisolone delivery routes, Tong et al. (2021) found intratympanic administration yielded superior hearing gains (21.6 dB) compared to oral (16.1 dB) or intravenous (14.3 dB) routes [55]. Similarly, two studies demonstrated improved outcomes with intratympanic and combination steroid–HBOT treatments, respectively [56,57]. Lopez-Campos et al. (2015) further explored mineralocorticoid therapy, showing greater hearing improvement than glucocorticoids or placebo, possibly due to enhanced ionic balance in the inner ear [58].

Several adjunctive therapies have also been investigated for their synergistic potential with steroids. Improved hearing outcomes with a combination of inhaled hydrogen gas, glucocorticoids, and PGE1 in diabetic patients have been reported, as well as herbal therapies such as Doluperine, Ginkgo Biloba extract, and Radix Astragali demonstrated meaningful gains when paired with intratympanic or systemic steroids [59,60,61,62,63]. Hyperbaric oxygen therapy (HBOT) enhances cochlear oxygenation and has been widely studied in combination with corticosteroids, with multiple studies reporting hearing improvements sometimes exceeding 30 dB in some subgroups [4,57,64,65,68]. An additional benefit of ITS and HBOT is a reduction in tinnitus [2]. However, results have been inconsistent: Two studies found no added benefit from HBOT or ITS over systemic steroids alone, while another observed benefits of HBOT in younger patients and ITS in those with profound hearing loss [5,66,67]. Despite challenges such as cost, accessibility, and potential side effects, the synergistic use of ITS, HBOT, and systemic steroids may offer a promising approach for SSNHL management, particularly in therapy-refractory cases.

### 3.5. Hematologic-Based Therapy

Table 5 summarizes eleven studies investigating hematologic-based therapy for SSNHL treatment. These interventions target vascular, immune, and microcirculatory mechanisms.

Platelet-rich plasma (PRP) therapy promotes nerve regeneration and reduces inflammation. Kanaujia et al. (2023) reported that 85.2% of patients with acute SNHL experienced complete or partial recovery following intratympanic PRP injections [69]. Similarly, hemorheological strategies such as fibrinogen/LDL apheresis and rheopheresis demonstrated improvements in patients unresponsive to corticosteroids by enhancing cochlear microcirculation and reducing plasma viscosity [70,71]. Oya et al. (2016) showed higher recovery rates in profound hearing loss patients treated with defibrinogenation therapy [72]. Sodium enoxaparin also showed comparable efficacy to corticosteroids, particularly when used in combination with venovenous hemofiltration [73].

**Table 5:** Hematologic-based therapy.

Study (Author)	Treatment	Proposed Clinical Use	Phase	Study Design	Population	Adverse Events
Kanaujia et al., 2023 [69]	IT platelet-rich plasma (PRP)	Acute SNHL	—	Prospective hospital-based interventional study	Total: 70 Age Range: 1–45 years	Transient dizziness (41.4%), pain (61.4%) post-injection
Canis et al., 2012 [70]	IV fibrinogen/LDL apheresis	SSNHL	—	Retrospective cohort	Total: 217 Mean Age: 54.1 years (range: 18–84 years)	Adverse events not reported
Oya et al., 2016 [72]	IV Batroxobin (defibrinogenation therapy)	SSNHL	—	Retrospective comparative study	Total: 116 Treatment: 59 Control: 57 Mean Age: Treatment: 59.2 years Control: 57.3 years	Adverse events not reported
Mösges et al., 2008 [71]	IV rheopheresis	Idiopathic SSNHL	—	Prospective, multicenter RCT	Total: 193 Rheopheresis: 94 Control: 99 Mean age: Rheopheresis: 45.3 years Control: 44.9 years	10 total; 2 severe events—allergic reaction with complete recovery; transient hypotension, nausea, dizziness.
Mora et al., 2006 [73]	IV sodium enoxaparin and venovenous hemofiltration	SSNHL with or without tinnitus	—	RCT	Total: 20 Treatment: 10 Control: 10 Age Range: 18–65 years	No adverse events reported by study authors
Zhai et al., 2013 [74]	Oral gushen pian	SNHL, tinnitus	Phase II	Double-blind, placebo RCT	Total: 120 Treatment: 40 Control: 40 Simple Treatment: 40 Age Range: 17–64 years	No adverse events reported by study authors
Klemm et al., 2007 [75]	IV Hydroxyethyl starch (HES) 130/0.4	Idiopathic SSNHL	Phase II	Double-blind, placebo, multicenter, dose-finding RCT	Total: 210 Group H (750 mL/d): 31 Group M (45 g/d): 31 Group L: (30 g/d): 35 Group G (15 g/d): 31 Mean Age: 46 years (range: 18–75 years)	Two serious AEs occurred (anaphylactic reaction and known arrhythmia recurrence), but neither was definitively linked to the study drug. Mild AEs included pruritus, headaches, and nausea
Kostal et al., 2017 [76]	IV & IT rheopheresis (apheresis) & MicroWick (IT dexamethasone)	SISHL refractory to systemic corticosteroids	—	Open-label, prospective, observational study	Total: 106 Rheopheresis: 33 MicroWick: 19 Control: 54 Mean Age: Rheopheresis: 58 years. MicroWick: 54 years. Control: 53 years	Rheopheresis: Transient nausea, 1 case of hypotension MicroWick: Persistent tympanic membrane perforation, need for surgical intervention, progression to deafness in 2 patients
Feng et al., 2022 [77]	IV alprostadil + HBOT	SSNHL	—	RCT	Total: 104 Alprostadil: 52 Alprostadil + HBOT: 52 Mean Age: 44.6 years (range: 20–59 years)	Adverse events not reported
Ahn et al., 2006 [78]	IV lipo-prostaglandin E + methylprednisolone	SSNHL in patients with type 2 diabetes mellitus	—	Prospective, double-blind RCT	Total: 270 Diabetic: 66 Non-Diabetic: 204 Mean Age: Diabetic: 59.9 years Non-Diabetic: 46.9 years	Vascular pain, headache, and flushing
Mora et al., 2005 [79]	IV sodium enoxaparin (low molecular weight heparin)	Immune-mediated SNHL with a history of autoimmune activity and alterations in immune blood parameters	—	Randomized, placebo-controlled study	Total: 30 Treatment: 15 Placebo: 15 Age Range: 20–65 years	No adverse events reported by study authors

Other novel approaches include Gushen Pian treatment, a traditional Chinese medicine shown to improve hearing outcomes, and hydroxyethyl starch (HES)-based hemodilution, which demonstrated modest benefits in early-stage SSNHL treatment [74,75]. An early phase 1 clinical trial is currently investigating the use of autologous blood monocyte vesicles to restore hearing by mitigating cochlear inflammation and enhancing cellular repair [22]. Another study found a 27% hearing improvement in the rheopheresis treatment group but no significant improvement in the comparative MicroWick group [76]. Feng et al. (2022) reported a higher total effective rate of treatment and hearing recovery rate in the treatment group taking a combination of Alprostadil and HBOT compared to Alprostadil alone [77]. However, the efficacy of these hematologic-based interventions varies depending on individual patient factors including age, initial hearing threshold, and comorbid conditions like diabetes, which influence therapeutic response. One study found that intravenous lipo-PGE1 with methylprednisolone significantly improved hearing recovery in type 2 diabetic SSNHL, while another reported that intravenous enoxaparin improved high-frequency hearing and tinnitus in immune-mediated SNHL patients [78,79].

### 3.6. Pathway Modulator Therapy

Table 6 summarizes four studies that investigated pathway modulator agents. Table 2 summarizes an additional eight pathway modulator agents in ongoing or recently completed clinical trials. Several FDA-approved drugs are currently under investigation for potential repurposing as treatments for SNHL, leveraging their established safety profiles. Statins, commonly used to lower high serum cholesterol levels, have demonstrated otoprotective properties in preclinical models of noise- and cisplatin-induced ototoxicity [80]. As such, statins are now under clinical evaluation for idiopathic SSNHL and cisplatin-induced hearing loss [23,24]. Topiramate, typically used for migraine prophylaxis, is being tested for its standalone efficacy in SSNHL, while donepezil, an Alzheimer’s medication, is under investigation for its ability to facilitate cortical reorganization in cochlear implant users [25,26]. Other repurposed agents include sirolimus, studied in Pendred syndrome for reducing hearing loss and vertigo, and the Liuwei Dihuang Pill (LDP), a traditional Chinese medicine in clinical trials for presbycusis [29,81].

In parallel, several novel therapeutics are in development targeting diverse molecular pathways. ACOU085, a Kv7.4 potassium channel modulator, is being tested for protection against cisplatin-induced hearing loss [27]. SENS-401, a 5-HT3 receptor antagonist and calcineurin inhibitor, was investigated as a potential otoprotectant from cisplatin-induced ototoxicity and also its ability to preserve residual hearing during cochlear implant surgery [82]. NS101, a novel aglycosylated monoclonal antibody targeting FAM19A5, is in trials for use as salvage therapy in SSNHL patients unresponsive to steroids [28]. AM-111, a c-Jun N-terminal kinase (JNK) inhibitor delivered in hyaluronic acid gel, has shown promise in preventing apoptosis of stress-injured hair cells and spiral ganglion neurons after impulse noise trauma [83,84]. Lastly, AC102 is in phase II trials for idiopathic SSNHL and may have a bimodal effect, both preventing noise-induced outer hair cell loss and reducing inner hair cell synaptopathy [30]. Overall, these innovative approaches will expand the pharmacologic options to treat SNHL and enhance understanding of the auditory pathway.

### 3.7. Regenerative Therapy

Table 7 summarizes three regenerative therapies investigated to either protect or stimulate cochlear hair cell growth. Insulin-like growth factor 1 (IGF-1), the primary anabolic effector hormone in the growth hormone axis, has shown potential as a salvage therapy for SSNHL cases unresponsive to corticosteroids [85]. Dave et al. (2021) further suggested that IGF-1 reverses SNHL by inducing transdifferentiation of supporting cells into hair cells and prevents apoptosis of hair cells via modulation of the PI3K/Akt pathway [86]. Another approach involves Notch signaling inhibition through gamma-secretase inhibitors like LY3056480. Although this agent did not meet its primary endpoint in a clinical trial, some participants experienced improvements in pure-tone audiometry and speech perception in noise [87].

Emerging stem cell-derived therapies are also under investigation. VSF1.01, an extracellular vesicle-enriched secretome fraction, is being evaluated for its ability to preserve residual hearing in cochlear implant recipients [31]. Prior work by Warnecke et al. (2021) demonstrated that vesicles derived from allogenic umbilical cord mesenchymal stem cells exhibit anti-inflammatory effects in the inner ear [88]. Although regenerative therapies are promising, further research is needed to overcome ethical, logistical, and biological challenges in translating these strategies into clinical care.

### 3.8. Gene Therapy

Genetic factors play a significant role in SNHL, with over 150 genetic variants currently implicated in its pathogenesis [89]. Among these, mutations in the OTOF gene, which encodes otoferlin, are a leading cause of congenital bilateral severe-to-profound hearing loss. Table 8 summarizes a clinical study by Lv et al. (2024), in which six children received a single cochlear injection of AAV1-hOTOF, identified as RRG-003, resulting in a 40–57 dB reduction in auditory brainstem response thresholds in five participants [90,91]. Immediately following these encouraging results, multiple companies including Akouos (AAVAnc80-hOTOF), Regeneron Pharmaceuticals (DB-OTO), Eye & ENT Hospital of Fudan University (EH002), Otovia Therapeutics (OTOV101N+OTOV101C), and Sensorion (SENS-501), launched early-phase trials targeting OTOF-related SNHL and are summarized in Table 2 [32,33,34,35,36]. Recent clinical data for DB-OTO have shown early hearing improvement in patients with OTOF-related deafness, supporting continued investigation of OTOF gene replacement as a clinically advanced gene-therapy approach for inherited SNHL [92]. If these trials continue to be successful, gene therapies could be expanded to target many of the other genetic mutations responsible for hearing loss, offering the potential to improve the lives of many individuals with SNHL and advance the fields of audiology and neurotology.

## 4. Discussion

This scoping review summarizes the pharmacologic agents investigated for SNHL over the past two decades. A total of 66 studies were categorized by mechanism of action. Antioxidants, corticosteroids, hematologic agents, and pathway modulators have shown potential in preventing or treating SNHL caused by cisplatin, aminoglycosides, noise-induced ototoxicity, and intraoperative cochlear implantation trauma [40,41,42]. These agents could reduce cochlear inflammation, oxidative stress, and vascular compromise, whereas regenerative strategies and gene therapies have attempted to restore auditory function in selected forms of SNHL [85,86,90]. Collectively, these studies underscore the multifactorial pathophysiology of SNHL and emphasize the need for multimodal interventions.

The therapies identified in this review should not be interpreted as interchangeable treatments for all forms of SNHL, because they address clinically distinct problems. Otoprotective agents, such as sodium thiosulfate, NAC, ebselen, ORC-13661, and ACOU085, are primarily intended to prevent treatment-related or exposure-related cochlear injury before irreversible damage occurs. SSNHL therapies, including systemic corticosteroids, intratympanic steroids, HBOT combinations, hematologic agents, and selected pathway modulators, are used after acute hearing loss has occurred and are influenced by time to treatment, baseline severity, and etiology. Cochlear implant-associated interventions, including antioxidant regimens and dexamethasone-eluting implants, focus on preserving residual hearing associated with intraoperative cochlear implantation trauma. Finally, regenerative and gene-based therapies target underlying cellular or genetic mechanisms.

### 4.1. Antioxidant, Steroid-Based Combination, Hematologic-Based, and Pathway Modulator Therapies

Antioxidant therapies reduce cochlear oxidative stress and protect hair cells from ototoxic insult. Antioxidants, including N-acetylcysteine, Coenzyme Q10, and combinations such as ACEMg (vitamins A, C, E, and magnesium), have demonstrated mixed efficacy, with results depending on the etiology and timing of intervention [43,44,45,50]. However, the recent FDA approval of PEDMARK marks a significant milestone in SNHL prevention, providing a clinically validated antioxidant therapy that paves the way for further advancements in otoprotective treatments [40,93,94].

Among emerging therapies for SSNHL, intratympanic steroids (ITS) delivery has remained a cornerstone as both a first-line and salvage therapy, highlighting the importance of the drug delivery route. Studies suggest that combining corticosteroids with HBOT improves hearing outcomes, particularly in younger or moderately affected patients [2,4,5,56,57,58,66,68]. Although there is no formal, universally accepted treatment plan for SSNHL, current recommendations focus on treating any underlying causes, performing a baseline evaluation with PTA hearing tests. Current evidence supports a protocol of systemic corticosteroids, ITS dexamethasone, and adjunctive HBOT in selected patients based on severity, timing, and treatment response [16,55]. There is an urgent need to refine treatment algorithms, especially for emerging therapies, to support pooled analyses and guide evidence-based recommendations [1,14,38]. Current AAO-HNSF guidelines emphasize prompt confirmation of SSNHL with audiometry, evaluation for retrocochlear pathology, patient counseling regarding the limitations of existing evidence, and consideration of intratympanic steroid therapy for incomplete recovery 2–6 weeks after symptom onset [95]. Although these guidelines provide a clinical framework for SSNHL management, the limited number of established pharmacologic options has prompted investigation into adjunctive and combination therapies.

Several studies have explored adjunctive and combination therapies to enhance treatment outcomes for SNHL. For example, herbal compounds such as Ginkgo biloba, Radix astragali, and Doluperine have shown potential synergistic effects when administered alongside conventional treatments [60,61,62,63,96]. Hematologic-based therapies, including platelet-rich plasma, fibrinogen/LDL apheresis, and hemorheological modulation, target microvascular and immune-mediated mechanisms. Evidence indicates these strategies can enhance cochlear perfusion, reduce plasma viscosity, and improve recovery in patients unresponsive to steroids [69,70,71,72,73,75,76,77,79]. Statins, topiramate, donepezil, and sirolimus are being investigated and repurposed for their otoprotective or neuroplasticity-enhancing effects [23,24,25,26,80,81,96].

Novel pathway modulating agents targeting potassium channels (ACOU085), JNK signaling (AM-111), 5-HT3/calcineurin pathways (SENS-401), and FAM19A5 (NS101) demonstrate a precision approach aimed at preserving hair cells, synaptic integrity, and auditory nerve function [27,28,82,83,84]. Although early-phase data for these studies is promising, efficacy remains variable and influenced by patient-specific factors such as age, comorbidities (e.g., diabetes), and baseline hearing thresholds [55,78].

### 4.2. Regenerative and Gene Therapies

Recent advances in regenerative medicine and gene therapy may meaningfully change the treatment landscape for SNHL. Regenerative strategies using IGF-1, Notch inhibitors, or extracellular vesicle-based therapies provide a potential route for cochlear repair, either by promoting hair cell transdifferentiation or mitigating apoptosis [85,86,87,88]. Despite ethical concerns surrounding stem cell application, regenerative therapy offers a compelling potential to restore hearing [7].

Ongoing and recently completed clinical trials are assessing the safety and efficacy of *OTOF* gene replacement in children with congenital OTOF-related deafness [32,33,34,35,36,90,91]. Lv et al. reported early human clinical results using AAV1-hOTOF gene therapy for DFNB9, establishing an important first-in-human milestone for congenital deafness gene therapy [90]. More recently, Jiang et al. reported on a multicenter 2.5-year follow-up data for AAV1-hOTOF therapy in OTOF-related deafness. The study found hearing recovery in 90% of treated participants, sustained auditory brainstem response improvement, and no dose-limiting toxicities [97].

With over 150 genetic variants already identified to be associated with SNHL, OTOF gene therapy holds promise as a pioneering treatment for genetically related SNHL [89,98]. Early-phase trials demonstrate safety and encouraging auditory improvements, suggesting that precision medicine approaches may become a cornerstone for genetically defined SNHL. However, current clinical gene-therapy evidence is concentrated primarily in OTOF-related hearing loss, and the limited long-term data across broader populations and genetic targets demonstrates the importance of extended follow-up studies, real-world effectiveness research, and robust post-marketing surveillance frameworks. The death of Jesse Gelsinger in a 1999 adenoviral-vector gene-therapy trial remains an important reminder that human gene-therapy studies require rigorous vector safety assessment, transparent informed consent, careful patient selection, and long-term surveillance [99].

### 4.3. Gaps and Limitations

This review excluded numerous animal studies, particularly regenerative therapies, as well as emerging technologies from private biotech companies, such as gene therapies, because they did not meet the eligibility criteria. Additionally, novel therapies that failed to meet primary objectives in clinical trials were omitted in order to emphasize the most viable treatment candidates. These exclusions highlight the broad range of emerging interventions not captured in this review.

Despite notable advances in pharmacologic and biologic therapies for SNHL, critical gaps remain. Many existing studies are constrained by small sample sizes, heterogeneous methodologies, short follow-up durations, and variable outcome measures, limiting cross-study comparisons and generalizability [1,3,11]. Advancing this field will require increased investment in translational research, improved infrastructure for multicenter collaboration, and strategies to overcome barriers to commercialization and delivery of novel gene therapies. Future research should emphasize robust, multicenter randomized controlled trials, stratified by etiology and severity [16].

### 4.4. Emerging Therapies and Future Directions

Hearing aids and cochlear implants have provided substantial benefits and filled a gap where pharmacologic interventions were largely absent [12,13]. This review identifies multiple pharmacotherapeutics currently under investigation in clinical trials to prevent and treat SNHL. However, this field lacks comparable breakthroughs involving monoclonal antibodies, gene editing platforms like CRISPR-Cas9, and advanced stem cell applications [7]. Continued investment in SNHL pharmacotherapy could have a substantial clinical and public health impact. During the original search window, PEDMARK was the only FDA-approved pharmacotherapy identified for the prevention of treatment-related SNHL [39]. Since completion of the search, Otarmeni (lunsotogene parvec-cwha), previously studied as DB-OTO within the clinical trial list, received FDA accelerated approval for OTOF-associated severe-to-profound or profound SNHL, expanding the landscape to include an approved gene-based therapy for a genetically derived form of SNHL [33,100].

A critical area for future research is drug delivery. The blood–labyrinth barrier (BLB) is a barrier between the blood vessels and the fluids of the inner ear [101]. This BLB naturally provides protection against ototoxic factors but may also be the root cause of poor response to previous therapeutic medications. This suggests that some clinical trials and medications may have been unsuccessful not because they were incapable of treating SNHL, but rather because the drug delivery method was insufficient. Even a relatively simple method, called cochlear pumping, demonstrated a consistent and uniform drug distribution along the entire length of the intact cochlea after an intratympanic injection [102]. Recent research also suggests that there is a bidirectional fluid exchange occurring between the cochlea and the CSF [98]. Thus, medication delivered intra-cisternal may be a direct method of drug delivery, but it also comes with a high risk of infection and brain/brainstem injury. Mastering these delivery strategies may unlock the full potential of existing and future therapies for SNHL.

## 5. Conclusions

This scoping review provides a structured overview of the pharmacologic treatments for sensorineural hearing loss (SNHL) based on published studies and clinical trial records from the past two decades. Interventions were categorized by mechanism of action to provide clinicians and researchers with a clear overview of existing and emerging therapeutic options, including antioxidant, steroid-based combination, hematologic-based, pathway modulator, regenerative, and gene therapies. The review also highlights gaps in the literature and identifies priorities for future research. Although several therapies have shown promise in selected clinical contexts, important limitations remain. Future research should address heterogeneous study designs, small sample sizes, inconsistent outcome measures, and limited long-term follow-up through collaborative, large-scale, multicenter studies. Continued innovation and investment will be critical in shaping a future in which pharmacologic therapies can more effectively prevent, treat, or potentially reverse hearing loss.

## Figures and Tables

**Table 1:** Studied agents. This table summarizes all studied agents organized by mechanism of action. Asterisks (*) indicate pharmacotherapeutic agents that are being evaluated in ongoing or recently completed clinical trials, which are summarized separately in Table 2.

Mechanism of Action	Studied Agents
Antioxidant Therapy	− Sodium Thiosulfate (STS); PEDMARK − Oral N-acetylcysteine (NAC) − IV NAC + IT Dexamethasone − Oral Coenzyme Q10 (Q-TER) − Q-TER + vitamin E − Oral Rebamipide +Vitamin C − High-dose IV vitamin C + systemic steroids − Oral ACEMg (vitamin A, C, & E + magnesium) − Oral SPI-1005 * − Oral ORC-13661 *
Steroid-Based Combination Therapy	− IT Dexamethasone − IT Prednisolone − HBOT + Oral Prednisone − Oral/IV/IT methylprednisolone − Oral mineralocorticoid vs. glucocorticoid − Inhaled Hydrogen gas − Oral Doluperine + IT Dexamethasone − Oral Ginkgo biloba extract + Tab Neurobion Forte − Oral Ginkgo biloba extract + IV methylprednisolone − IV Radix Astragali + Dexamethasone − HBOT + IT Prednisolone − HBOT + IT Steroids + Oral Systemic Steroids − HBOT + IT Dexamethasone − Oral Prednisolone + HBOT + IT Dexamethasone − Cochlear implant w/slow-release Dexamethasone *
Hematologic-Based Therapy	− IT Platelet-rich Plasma (PRP) − IV Fibrinogen/LDL Apheresis − IV Batroxobin − IV Rheopheresis − IV sodium enoxaparin + venovenous hemofiltration − Oral Gushen Pian − IV Hydroxyethyl starch − IV/IT Rheopheresis + IT Dexamethasone − HBOT + IV Alprostadil − IV Lipo-prostaglandin E1 + methylprednisolone − IV sodium enoxaparin − Autologous blood monocyte vesicles *
Pathway Modulator Therapy	− Oral Sirolimus − Oral SENS-401 − IT AM-111 − IV NS101 * − Transtympanic ACOU085; Bimokalner * − Liuwei Dihuang Pill (LDP) * − Oral statins + Oral methylprednisolone * − Atorvastatin * − IT AC102 * − Oral Topiramate * − Oral cholinergic enhancement with Donepezil *
Regenerative Therapy	− IT IGF-1 − IT IGF-1 + gelfoam − IT LY3056480 (Gamma-secretase inhibitor) − IC Extracellular vesicular-enriched secretome fraction *
Gene Therapy	− IC AAV1-hOTOF − IC DB-OTO * − IC EH002 * − IC AAVAnc80-hOTOF (AK-OTOF) * − IC OTOV101N + OTOV101C * − IC SENS-501 *

**Table 2:** Summary of ongoing and recently completed clinical trials. This table lists the 18 ongoing or recently completed clinical trials identified in this review. Trial records were not treated as equivalent to peer-reviewed published efficacy evidence and were summarized separately to characterize the current investigational pipeline.

Mechanism of Action	Identifier & Treatment	Proposed Clinical Use	Phase	Study Design	Population Age	Sponsor
Antioxidant Therapy	NCT06340633 Oral SPI-1005; Ebselen [19]	Adults receiving a cochlear implant	Phase II	Randomized, double-blind, placebo-controlled	18 years and older	Sound Pharmaceuticals
Antioxidant Therapy	NCT05730283 Oral ORC-13661 [20]	Amikacin-induced ototoxicity prophylaxis in nontuberculous mycobacteria	Phase II	Double-blind, placebo RCT	18 years to 80 years	Kevin Winthrop, Oregon Health and Science University
Steroid-Based Combination Therapy	NCT06424262 Cochlear implant (CI622D) that slowly releases dexamethasone [21]	SNHL	—	Prospective, blinded RCT	18 years and older	Cochlear
Hematologic-Based Therapy	NCT06707389 Autologous blood monocyte vesicles [22]	SSNHL	Early Phase I	Single-arm, open-label, early-stage efficacy, and safety trial	Between 18 and 65 years	Sun Yat-Sen Memorial Hospital of Sun Yat-Sen University
Pathway Modulator Therapy	NCT04826237 Oral statins and oral methylprednisolone [23]	ISSNHL diagnosed within 14 days of symptom onset.	Phase IV	Randomized controlled trial.	18 years to 80 years	Northwestern University
Pathway Modulator Therapy	NCT06553157 Atorvastatin [24]	Patients with solid tumors scheduled to receive platinum chemotherapies	Phase I/II	Randomized, placebo-controlled, parallel assignment with no masking (open label)	18 years to 75 years	Minia University
Pathway Modulator Therapy	NCT05403229 Oral topiramate [25]	SSNHL	Phase II	Randomized, controlled, potentially double-blind	20 years and older	Chang Gung Memorial Hospital
Pathway Modulator Therapy	NCT05438264 Oral cholinergic enhancement with donepezil [26]	Cochlear Implant Users	Early Phase I	randomized, double-blind, placebo-controlled trial.	18 years to 100 years	Vanderbilt University Medical Center
Pathway Modulator Therapy	NCT06521190 Transtympanic ACOU085; Bimokalner [27]	Prevention of cisplatin-induced hearing loss in testicular cancer patients	Phase II	Randomized, double-blind, placebo-controlled, multicenter split-body trial	18 years to 45 years	Acousia Therapeutics GmbH
Pathway Modulator Therapy	NCT06249919 IV NS101; anti FAM19A5 antibody [28]	SSNHL	Phase 1/2	Prospective, randomized, double-blind, placebo-controlled, multicenter, dose-escalation	19 years to 65 years	Neuracle Science Co., LTD.
Pathway Modulator Therapy	NCT05125081 Liuwei Dihuang Pill (LDP) [29]	Presbycusis associated with renal deficiency	Phase II	Randomized, double-blind, placebo-controlled assignment	65 to 75 years	Shanghai Jiao Tong University School of Medicine
Pathway Modulator Therapy	NCT05776459 IT AC102 [30]	Idiopathic SSNHL	Phase II	Randomized, double-blind, placebo-controlled, multicenter study	18 years to 85 years	AudioCure Pharma GmbH
Regenerative Therapy	NCT06545175 Intracochlear (IC) application of extracellular vesicle-enriched secretome fraction (VSF1.01) [31]	Meets local candidacy criteria for cochlear implantation	Phase I/II	Open-label monocentric	18 years and older	Hannover Medical School
Gene Therapy	NCT05821959 IC injection of AAVAnc80-hOTOF (OTOF) [32]	OTOF mutation in both alleles	Phase I/II	Randomized, open-label, dose-escalation, multicenter	Participants of any age	Akouous, Inc.
Gene Therapy	NCT05788536 IC injection of DB-OTO; adeno-associated virus (AAV) [33]	OTOF mutation in both alleles	Phase I/II	Non-randomized open-label trial	Up to 17 years	Regeneron Pharmaceuticals
Gene Therapy	NCT06722170 IC injection of EH002 [34]	DFNB9 congenital deafness	—	Non-randomized open-label trial	6 months and older	Eye & ENT Hospital of Fudan University
Gene Therapy	NCT05901480 IC injection of OTOV101N and OTOV101C; adeno-associated virus (AAV) [35]	OTOF mutations (DFNB9)	—	Investigator-initiated, open-label trial	1 year and older	Otovia Therapeutics
Gene Therapy	NCT06370351 IC injection of SENS-501 [36]	Children with severe to profound hearing loss due to otoferlin gene mutations	Phase I/II	Multicenter, adaptive, open-label, non-randomized, dose-escalation, and expansion study	6 months to 31 months	Sensorion

**Table 3:** Antioxidant therapies.

Study (Author)	Treatment	Proposed Clinical Use	Phase	Study Design	Population	Adverse Events
Freyer et al., 2017 [40]	Sodium Thiosulfate (STS, PEDMARK)	Cisplatin-induced ototoxicity prophylaxis	Phase III	Multicenter, open-label RCT	Total: 125 Treatment: 61 Control: 64 Age Range: 1–18 years	None, probably or definitely related to STS
Tokgoz et al., 2011 [41]	Oral N-acetylcysteine (NAC)	Aminoglycoside and vancomycin-induced ototoxicity prophylaxis	—	RCT	Total: 60 NAC: 30 Control: 30 NAC: 45.0 years (mean age) Control: 49.9 years (mean age)	Adverse events not reported
Kocyigit et al., 2015 [42]	Oral NAC	Amikacin-induced ototoxicity prophylaxis	—	RCT	Total: 46 NAC: 23 Control: 23 NAC: 46.6 years (mean age) Control: 49.5 years (mean age)	No adverse events reported by study authors
Lin et al., 2010 [43]	Oral NAC	Prevention of noise-induced temporary threshold shifts (TTS) in occupationally noise-exposed individuals	—	Prospective, double-blind, crossover trial	Total: 83 Sequence 1 (NAC first): 53 Sequence 2 (placebo first): 28 Sequence 1: 40.0 years (mean age) Sequence 2: 42.1 years (mean age)	Adverse events not reported
Chen et al., 2017 [44]	Intravenous (IV) NAC	Sudden SNHL	—	Comparative study	Total: 70 Group A (NAC only): 35 Group B (corticosteroids and plasma expander): 35 Group A: 44 years (mean age) Group B: 48 years (mean age)	No adverse events reported by study authors
Bai et al., 2021 [45]	Oral NAC and intratympanic (IT) dexamethasone	Refractory sudden SSNHL	—	Open-label RCT	Total: 41 NAC + Dexamethasone: 18 Dexamethasone-only: 23 Mean Age: 38.5 years (range: 14–60 years)	Adverse events not reported
Salami et al., 2010 [46]	Oral Coenzyme Q10 (Q-TER)	Presbycusis	—	Double-blind, placebo RCT	Total: 60 Group A (Q-TER): 20 Group B (Vitamin E): 20 Group C (Placebo): 20 Group A: 69.8 years (mean age) Group B: 70.1 years (mean age)	Adverse events not reported
Guastini et al., 2011 [47]	Q-TER and Vitamin E	Presbycusis	—	Double-blind, placebo RCT	Total: 60 Group A (Q-TER 160 mg daily): 20 Group B (Vitamin E 50 mg daily): 20 Group C (Placebo): 20 Age Range: 65–76 years	Adverse events not reported
Ahn et al., 2010 [48]	Q-TER	SSNHL	—	Controlled, prospective study	Total: 120 Treatment: 60 Control: 60 Treatment: 45.4 years (mean age) Control: 49.5 years (mean age)	Adverse events not reported
Takumida et al., 2005 [49]	Oral Rebamipide and Vitamin C	Presbycusis	—	Pilot study without a control group	Total: 23 Mean Age: 77.1 years (range: 70–89 years)	No adverse events reported by study authors
Kang et al., 2013 [50]	High-dose IV vitamin C (HDVC) and systemic steroids	Idiopathic sudden sensorineural hearing loss (ISSNHL) diagnosed within 14 days of onset	—	Prospective, single-blind, RCT	Total: 72 Treatment (HDVC): 36 Control: 36 Treatment: 52 years (mean age) Control: 50.3 years (mean age)	No adverse events reported by study authors
Scheper et al., 2020 [51]	Oral ACEMg (vitamins A, C, & E + magnesium)	Residual hearing preservation in CI	Phase II	Double-blind, placebo-controlled	Total: 51 Treatment: 25 Control: 24 Mean Age: 55.47 years	Mild ear pain, vertigo, tympanic perforation

**Table 4:** Steroid-based combination therapy.

Study (Author)	Treatment	Proposed Clinical Use	Study Design	Population	Adverse Events
Dispenza et al., 2013 [6]	IT dexamethasone	Idiopathic SSNHL that did not respond to initial systemic treatment	Prospective Study	Total: 46 Treatment: 36 Control: 10 Mean Age: 49.4 years (range: 17–74 years)	No adverse events reported by study authors
Filipo et al., 2013 [8]	IT prednisolone	Idiopathic SSNHL with a flat audiogram	Prospective, triple-blind, placebo RCT	Total: 50 Treatment: 25 Placebo: 25 Mean Age: 50.4 years (range: 15–85 years)	Mild pain at the injection site and transient vertigo.
Zjawiony et al., 2020 [56]	Oral prednisone and HBOT	Idiopathic SSNHL	Prospective study	Total: 40 Group I (Combination): 24 Group II (Only Pharm): 16 Mean Age: 52.4 years (range: 33–77 years)	Adverse events not reported
Tong et al., 2021 [55]	Oral methylprednisolone, IV methylprednisolone, IT methylprednisolone	Idiopathic SSNHL	Prospective RCT	Total: 90 Group I (Oral): 30 Group II (IV): 30 Group III (IT): 30 Mean Age: Group I: 42.9 years Group II: 44.9 years Group III: 40.2 years	Group III: 6 patients experienced transient dizziness during treatment. Some patients reported tolerable pain during injection
López-Campos et al., 2015 [58]	Oral mineralocorticoid (Fludrocortisone) vs. glucocorticoid (Deflazacort)	Idiopathic SNHL	Prospective, non-double-blind, RCT	Total: 90 Group A (Fludrocortisone): 26 Group B (Deflazacort): 22 Group C (Nimodipine): 22 Group D (Placebo): 20 Age Range: 19–71 years	Blood pressure changes in corticosteroid groups (3 in Deflazacort and 5 in Fludrocortisone groups), normalized within 2 weeks using a thiazide diuretic (Hidrosaluretil).
Okada et al., 2022 [59]	Inhaled hydrogen gas (H_2_)	Idiopathic SSNHL	Double-blind, placebo RCT	Total: 65 Treatment: 31 Control: 34 Mean Age: 60 years	No adverse events reported by study authors
Tajdini et al., 2023 [60]	Oral Doluperine^®^ (curcumin, piperine, gingerol) and IT dexamethasone	SSNHL in diabetic patients	Triple-blind, placebo RCT	Total: 51 (All 3 groups had IT steroids) Group A (Control, 2 Placebos): 17 Group B (Doluperine^®^ + Placebo: 17 Group C (Doluperine^®^ x2): 17 Mean Age: 55.5–58.9 years (range: 20–65 years)	No adverse events reported by study authors
Sharma et al., 2021 [61]	Oral ginkgo biloba extract and Tab Neurobion Forte (Vitamin B Complex)	SNHL and tinnitus	Randomized prospective clinical study	Total: 60 Group A (Ginkgo biloba + Conventional): 30 Group B (Conventional only): 30	Adverse events not reported
Koo et al., 2015 [62]	Oral ginkgo biloba extract (EGb761) and IV methylprednisolone	Idiopathic SSNHL	Double-blind, placebo RCT	Total: 56 (Both groups had methylprednisolone) Treatment: 26 Placebo: 30 Age Range: 20–70 years	Mild rash (1 participant), no severe events
Xiong et al., 2011 [63]	IV Radix Astragali (RA) with Dexamethasone	Sudden deafness	Prospective clinical study	Total: 92 Treatment (RA): 46 Control: 46 Mean Age: Treatment: 45.7 years Control: 44.2 years	Adverse events not reported
Filipo et al., 2012 [57]	HBOT with IT prednisolone	Severe and profound idiopathic sudden SNHL	Randomized pilot study	Total: 48 Group A (Severe; IVS + HBOT): 13 Group B (Profound; IVS + HBOT): 13 Group C (Severe; ITS + HBOT): 12 Group D (Profound; ITS + HBOT): 10 Mean Age: 41.7 years (range: 18–85 years)	Adverse events not reported
Cho et al., 2018 [4]	Oral systemic steroids, HBOT, and IT steroids	Severe-to-profound idiopathic SSNHL	Prospective RCT	Total: 58 Control Group (ITSI): 30 Study Group (ITSI+HBOT): 28 Mean Age: Control: 56.1 years Study: 53.8 years	Mild otalgia during HBOT (2 patients; resolved). No significant adverse effects reported
Lamm et al., 2016 [64]	HBOT and IT dexamethasone	Therapy-refractory SSNHL, including idiopathic, noise-induced, and barotrauma-induced cases	Prospective case series	Total: 7 Idiopathic: 4 Noise: 2 Barotrauma: 1 Mean Age: 42.4 years (range: 26–62 years)	No adverse events reported by study authors
Ajduk et al., 2023 [65]	HBOT and IT dexamethasone	Idiopathic SSNHL after failed systemic steroid therapy	Retrospective, non-randomized interventional cohort study	Total: 126 Group A (HBO): 35 Group B (ITS): 43 Group C (Control): 48 Mean Age: 59.4 years	No significant side effects reported. ITS is associated with rare tympanic perforation in other studies, though none occurred in this cohort
Naiboğllu et al., 2015 [66]	Oral systemic prednisone, HBOT, and IT dexamethasone	Salvage therapy for Idiopathic SSNHL	Prospective clinical study	Total: 58 Group A (systemic steroid+HBOT): 20 Group B (ITS + systemic steroid + HBOT): 38	ITS-associated: Pain, transient vertigo, and mild discomfort
Keseroğlu et al., 2020 [67]	Oral methylprednisolone, HBOT, and IT dexamethasone.	Idiopathic SSNHL	Retrospective clinical trial	Total: 96 Group A (systemic steroid): 32 Group B (systemic steroid + ITS): 32 Group C (systemic steroid + HBO): 32 Mean Age: Group A: 45.81 years Group B: 48.62 years Group C: 43.31 years	Adverse events not reported
Cvorovic et al., 2013 [5]	HBOT and IT dexamethasone	Idiopathic SSHL with failure of primary corticosteroid therapy (hearing gain < 10 dB)	Prospective RCT	Total: 50 HBO group: 25 IT DEX group: 25 Mean Age: HBO: 53.6 years IT DEX: 47.3 years	HBO: serous otitis media resolved conservatively. IT DEX: Mild ear pain during injections resolved with analgesics

**Table 6:** Pathway modulator therapy.

Study (Author)	Treatment	Proposed Clinical Use	Phase	Study Design	Population	Adverse Events
Fujioka et al., 2020 [81]	Oral Sirolimus (NPC-12T; mTOR inhibitor)	Pendred Syndrome/DFNB4	Phase I/IIa	Phase I/IIa, double-blind, placebo-controlled	Total: 16 Treatment: 12 Control: 4 Age Range: 7–50 years	Mild dermatitis, increased triglycerides
Braverman et al., 2024 [82]	Oral SENS-401 (5-HT3 antagonist + Calcineurin inhibitor)	Acute SSNHL	Phase IIb	Randomized, double-blind, placebo-controlled	Total: 115 SENS-401 29 mg group: 38 SENS-401 43.5 mg group: 39 Placebo group: 38 Age Range: 18–83 years	None significant
Suckfuell et al., 2014 [83]	IT AM-111 (JNK inhibitor)	Acute SSNHL	Phase II	Multicenter, double-blind, randomized, placebo-controlled	Total: 210 AM-111 Low-Dose: 68 AM-111 High Dose: 70 Placebo: 72 Age Range: 18–61 years	Mostly mild or moderate: ear discomfort, tinnitus, and transient hearing deterioration. Serious adverse events were rare and unrelated to treatment
Staecker et al., 2019 [84]	IT AM-111 (Brimapitide): A 31-amino acid peptide JNK inhibitor	Idiopathic SSNHL, particularly profound cases.	Phase III	Double-blind, randomized, placebo-controlled; 91-day follow-up	Total: 256 AM-111 0.4 mg/mL: 85 AM-111 0.8 mg/mL: 86 Placebo: 85 Mean Age: 46 years (range: 18–65 years)	Common events: Ear and labyrinth disorders (e.g., vertigo, ear pain). Severe adverse events were rare and mostly unrelated to treatment

**Table 7:** Regenerative therapy.

Study (Author)	Treatment	Proposed Clinical Use	Phase	Study Design	Population	Adverse Events
Nakagawa et al., 2014 [85]	IT IGF-1 (gelatin hydrogel) vs. dexamethasone	SSNHL refractory to systemic corticosteroids	—	Multicenter, RCT	Total: 120 IGF-1: 62 Dexamethasone: 58 Mean Age: 49.3 years (20 years or older)	IGF-1: Mild transient adverse events (e.g., otitis media, nausea). Dex: Persistent tympanic membrane perforation in 15.5% of cases
Dave et al., 2021 [86]	IT IGF-1 and gelfoam in round window niche	Ototoxic and idiopathic SSNHL	—	Single-center case series	Total: 40 Treatment: 20 Control: 20 Mean Age: 31.3 years (range: 13–63 years)	Pain (88%, resolved in ~3 days), dizziness (24%), headache (20%), 1 case of acute suppurative otitis media (ASOM) with tympanic perforation
Schilder et al., 2024 [87]	IT LY3056480 (Gamma-secretase inhibitor, GSI)	Mild-moderate SNHL	Phase I/IIa	Open-label, multiple-ascending dose	Total: 44 Age Range: 22–79 years	Dizziness in 13 participants (22%), headache and oropharyngeal pain in 5 participants each (8%), dysgeusia in 4 (7%), throat irritation in 2 (3%), and single cases (2%) of postural dizziness, hypoaesthesia, dry throat, skin rash, and acne

**Table 8:** Gene therapy.

Study (Author)	Treatment	Proposed Clinical Use	Phase	Study Design	Population	Adverse Events
Lv et al., 2024 [90]	IC AAV1-hOTOF (RRG-003); escalating doses	Genetic deafness (DFNB9; severe-to-profound hearing loss)	Phase 1	Open-label, single-arm, single-center trial	Total: 6 Age Range: 1–18 years	48 adverse events, 2 events of decreased neutrophil count in 1 participant

## Data Availability

Data are available upon reasonable request from the corresponding author.

## Abbreviations

The following abbreviations are used in this manuscript:

5-HT3	5-hydroxytryptamine type 3
AAV	adeno-associated virus
ABR	auditory brainstem response
ACEMg	vitamins A, C, E, and magnesium
AE	adverse event
BLB	blood–labyrinth barrier
CI	cochlear implant
CRISPR-Cas9	clustered regularly interspaced short palindromic repeats and CRISPR-associated protein 9
CSF	cerebrospinal fluid
DFNB9	autosomal recessive deafness 9
FDA	Food and Drug Administration
GPx	glutathione peroxidase
GSI	gamma-secretase inhibitor
HBOT	hyperbaric oxygen therapy
HDVC	high-dose vitamin C
HES	hydroxyethyl starch
IC	intracochlear
IGF-1	insulin-like growth factor 1
IRB	Institutional Review Board
ISSNHL	idiopathic sudden sensorineural hearing loss
IT	intratympanic
ITS	intratympanic steroids
IV	intravenous
JNK	c-Jun N-terminal kinase
LDL	low-density lipoprotein
LDP	Liuwei Dihuang Pill
mTOR	mammalian target of rapamycin
NAC	N-acetylcysteine
NCE	new chemical entity
OSF	Open Science Framework
OTOF	otoferlin gene
PGE1	prostaglandin E1
PI3K/Akt	phosphoinositide 3-kinase/protein kinase B
PRISMA-ScR	Preferred Reporting Items for Systematic Reviews and Meta-Analyses extension for Scoping Reviews
PRP	platelet-rich plasma
PTA	pure-tone audiometry
Q-TER	water-soluble coenzyme Q10 formulation
RA	Radix Astragali
RCT	randomized controlled trial
ROS	reactive oxygen species
SNHL	sensorineural hearing loss
SSNHL	sudden sensorineural hearing loss
STS	sodium thiosulfate
TTS	temporary threshold shift
WHO	World Health Organization

## Appendix A

**Table A1:** Search Strategy.

Component	Search Keywords
Population:	(“Sensorineural hearing loss” OR SNHL OR “Sudden Sensorineural Hearing Loss” OR “Noise-induced hearing loss” OR “Presbycusis” OR “cochlea” OR “spiral ganglion neurons” OR “ribbon synapses” OR “Ototoxic hearing loss” OR “Acoustic trauma” OR “Auditory neuropathy” OR “Mixed hearing loss” OR “Retrocochlear hearing loss”)
Intervention:	(“Pharmacotherapy” OR “Drug therapy” OR “Corticosteroids” OR “Antioxidants” OR “N-acetylcysteine” OR “Ebselen” OR “Stem cell therapy” OR “gene therapy” OR “FX-322” OR “BDNF” OR “Statin” OR “Atorvastatin” OR “SENS-401” OR “SPI-1005” OR “Valproic acid” OR “ATOH1 gene transfer” OR “γ-secretase inhibitors” OR “Follistatin” OR “FSK” OR “CHIR” OR “DZNep” OR “TTNPB”)
Study Type:	(“Clinical trial” OR “Approved drugs” OR “Investigational drugs”)
